# Microglia in the dorsal raphe nucleus plays a potential role in both suicide facilitation and prevention in affective disorders

**DOI:** 10.1007/s00406-017-0774-1

**Published:** 2017-02-22

**Authors:** Ralf Brisch, Johann Steiner, Christian Mawrin, Marta Krzyżanowska, Zbigniew Jankowski, Tomasz Gos

**Affiliations:** 1 Department of Forensic Medicine, Medical University of Gdańsk, ul. Dębowa 23, 80-204 Gdańsk, Poland; 2 Department of Psychiatry, Otto-von-Guericke-University, Magdeburg, Germany; 3 Institute of Neuropathology, Otto-von-Guericke-University, Magdeburg, Germany; 4 Department of Zoology/Developmental Neurobiology, Institute of Biology, Otto-von-Guericke-University, Magdeburg, Germany

**Keywords:** Postmortem, Suicide, Dorsal raphe nucleus, Microglia

## Abstract

An involvement of the central serotonergic system has constantly been reported in the pathogenesis of suicide. The dorsal raphe nucleus (DRN) is the main source of serotonergic innervation of forebrain limbic structures disturbed in suicidal behaviour, in which an abnormal microglia reaction seems to play a role. In our present study, the density of microglia immunostained for the HLA-DR antigen was evaluated in the DRN. These analyses were carried out on paraffin-embedded brains from 24 suicidal and 21 non-suicidal patients; among them, 27 depressed (15 major depressive disorder and 12 bipolar disorder) and 18 schizophrenia (9 residual and 9 paranoid) patients and 22 matched controls without mental disorders. Only the non-suicidal depressed subgroup revealed significantly lower microglial reaction, i.e., a decreased density of HLA-DR positive microglia versus both depressed suicide victims and controls. The effect was not related to antidepressant or antipsychotic medication, as the former correlated positively with microglial density in non-suicidal depressed patients, and the latter had no effect. Moreover, the comparison of these results with previously published data from our workgroup in the same cohort (Krzyżanowska et al. in Psychiatry Res 241:43–46, 4) suggested a positive impact of microglia on ribosomal DNA transcription in DRN neurons in the non-suicidal depressed subgroup, but not in depressed suicidal cases. Therefore, the interaction between microglia and neurons in the DRN may be potentially involved in opposite ways regarding suicide facilitation and prevention in the tested subgroups of depressed patients.

## Introduction

Disturbances of the central serotonergic system are implicated in a multifaceted way in suicidal behaviour (for reviews, see: [1, 2]), which has been proposed to be an independent mental disorder in the fifth edition of the Diagnostic and Statistical Manual of Mental Disorders—DSM V [3] in accordance with numerous neurobiological research data (for reviews, see: [1, 2]). However, differences in suicide neurobiology related to the main psychiatric diagnosis seem to be accentuated despite of diagnoses-overreaching phenomena specific for suicide [4, 5].

As revealed by neuropathological research on suicide, abnormalities in the serotonergic system may be structurally restricted to a specific brain region, the dorsal raphe nucleus (DRN), which affects brain circuits (for a review see: [6]). DRN neurons provide the major serotonergic innervation to the prefrontal cortex (PFC) [7–9], which plays a pivotal role in behavioural regulation. Limbic regions of the PFC (i.e., the anterior cingulate cortex and the orbitofrontal cortex), in turn, may reciprocally regulate DRN function through direct pyramidal input [10, 11] modulated by microglia in both healthy and disordered brain (for reviews, see: [12, 13]).

A number of postmortem studies revealed changes in the DRN of suicide victims [4, 5, 14–28]. Current research points to immune activation as a possible causal factor underlying the pathophysiology of suicidal behaviour (for reviews see: [12, 29]). The deteriorating impact of neuroinflammation on DRN neurons has been revealed in experimental conditions, which was paralleled by microglia activation [30, 31]. Our recent research by the AgNOR (argyrophilic nucleolar organising region) silver staining has suggested a decreased ribosomal DNA (rDNA) transcription in DRN neurons specific for suicide regardless of the main psychiatric diagnosis of affective disorders or schizophrenia [4]. However, neither postmortem nor neuroimaging studies of the DRN in mental disorders, among them suicidal behaviour, have yet addressed the issue of microglial reactivity in this structure.

Previously, we have hypothesized that microglia activation in forebrain regions (among them PFC regions) could constitute a diagnose-overreaching phenomenon specific for suicide [32], which has been also suggested by other studies [33]. Consequently, in this study of brains from the Magdeburg Brain Bank, we hypothesized microglia activation in the DRN in suicidal patients regardless of their established diagnosis of affective disorders or schizophrenia. Moreover, we hypothesized a deteriorating impact of microglia on the rDNA transcriptional activity in DRN neurons. We tested these hypotheses by the evaluation of microglia immunostained for the HLA-DR antigen, which is up-regulated in activated microglia compared to the constitutive expression level observed in the resting human microglia ([34–36]; for a review, see: [37, 38]). Subsequently, we evaluated correlations between microglial density and AgNOR parameters obtained recently in the same cohort [4]. We aimed at both basic research on the neurobiology of suicide and the verification of our hypothesis presented previously [32].

### Methods

#### Human brain tissue

Brains of controls (*n* = 22) and both suicidal (*n* = 24) and non-suicidal patients (*n* = 21) with established diagnoses of a depressive episode in major depressive disorder (MDD) and bipolar disorder (BD) (*n* = 27) or paranoid and residual schizophrenia (*n* = 18) according to DSM-IV criteria, and no history of substance abuse, were obtained in accordance with existing EU law regulations from the Magdeburg Brain Bank (Germany). Most of investigated cases overlapped with those presented previously according to the differences in rDNA transcriptional activity in DRN neurons between schizophrenia and depression [39] and between suicidal and non-suicidal patients from both diagnostic groups [4]. The study has been approved by the local ethics committees of the University of Magdeburg and the Medical University of Gdańsk as performed in accordance with the ethical standards laid down in the Declaration of Helsinki of 1989. Demographic and clinical data are summarized in Table 1.

**Table 1:** Detailed diagnostic and demographic data and the microglia density values in the dorsal raphe nucleus (rounded off to the whole values) of suicidal (*n* = 24) and non-suicidal patients (*n* = 21), and healthy control subjects (*n* = 22)

Case ID	Diagnosis (DSM-IV)	Sex	Age [year]	PMI [h]	Illness duration [year]	CPZ [mg]	AD [mg]	Microglia density (cells/mm^2^)	Cause of death
1	Sz, paranoid	f	49	72	2	300	–	194	Jumping from the high place
2	Sz, paranoid	f	63	72	41	–	–	364	Hanging
3	Sz, paranoid	f	52	24	28	–	–	477	Self-harm by drowning
4	Sz, paranoid	f	55	48	6	–	–	81	Self-poisoning (overdose of medication)
5	Sz, paranoid	m	34	5	5	–	–	446	Hanging
6	Sz, paranoid	m	38	24	16	505	–	280	Self-strangulation
7	Sz, paranoid	m	27	24	5	150	–	39	Hanging
8	Sz, residual	m	76	12	26	–	–	61	Self-strangulation
9	Depressed, MDD	f	39	48	7	–	93	90	Self-poisoning (overdose of medication)
10	Depressed, MDD	f	47	24	11	–	–	218	Self-poisoning (overdose of medication)
11	Depressed, MDD	f	46	48	11	109	124	237	Hanging
12	Depressed, MDD	f	68	96	4	–	–	588	Hanging
13	Depressed, MDD	f	26	22	–	–	–	130	Jumping from the high place
14	Depressed, MDD	m	35	15	2	–	–	190	Self-harm by sharp object (cut wound of the forearm)
15	Depressed, MDD	m	36	42	1	–	–	170	Hanging
16	Depressed, MDD	f	60	24	–	–	–	123	Hanging
17	Depressed, BD	m	57	48	–	–	–	61	Hanging
18	Depressed, BD	m	60	24	–	–	–	144	Self-strangulation
19	Depressed, BD	f	46	4	13	327	133	90	Self-poisoning (overdose of medication)
20	Depressed, BD	f	59	72	24	–	112	278	Self-poisoning (overdose of medication)
21	Depressed, BD	m	47	24	9	–	20	202	Self-harm by sharp object (stab wound)
22	Depressed, BD	m	42	17	16	47	95	361	Hanging
23	Depressed, BD	m	53	24	–	–	–	282	Self-strangulation
24	Depressed, MDD	f	53	48	–	–	–	326	Hanging
Suicidal patients : ratio/median (*q1;q3*)		11m/13f	48 (38.5; 58)	24 (23; 48)	10 (5; 16)	225 (109; 327)	103.5 (93; 124)	198 (107; 304)	
25	Sz, paranoid	m	45	72	20	740	–	60	Acute respiratory failure (bronchopneumonia)
26	Sz, residual	m	46	48	18	846	–	838	Acute cor pulmonale (pulmonary embolism)
27	Sz, residual	m	51	48	28	758	–	480	Toxic shock syndrome (ileus, peritonitis)
28	Sz, residual	m	57	72	23	–	–	135	Sudden cardiac death
29	Sz, residual	f	64	12	0	–	–	736	Acute cor pulmonale (pulmonary embolism)
30	Sz, residual	m	48	48	32	–	–	180	Acute respiratory failure
31	Sz, residual	m	58	24	25	–	–	321	Cardiorespiratory failure
32	Sz, residual	m	54	48	34	–	–	348	Cardiorespiratory failure
33	Sz, residual	f	54	24	18	600	–	35	Acute cor pulmonale (pulmonary embolism)
34	Sz, paranoid	f	38	12	8	–	–	251	Sudden cardiac death
35	Depressed, MDD	f	63	17	2	–	50	116	Acute cor pulmonale (pulmonary embolism)
36	Depressed, MDD	f	61	70	11	111	30	85	Sudden cardiac death
37	Depressed, MDD	f	41	20	2	501	17	186	Acute cor pulmonale (pulmonary embolism)
38	Depressed, MDD	f	62	72	11	110	–	95	Acute cor pulmonale (pulmonary embolism)
39	Depressed, MDD	f	60	14	10	109	52	29	Acute respiratory failure (bronchopneumonia)
40	Depressed, MDD	m	39	14	2	280	–	97	Acute cor pulmonale (pulmonary embolism)
41	Depressed, BD	m	39	56	14	221	–	78	Coronary failure
42	Depressed, BD	m	69	48	26	–	–	57	Acute cor pulmonale (pulmonary embolism)
43	Depressed, BD	m	69	24	18	–	–	112	Acute respiratory failure (bronchopneumonia)
44	Depressed, BD	f	52	24	–	–	–	171	Acute cor pulmonale (pulmonary embolism)
45	Depressed, BD	f	65	52	25	117	93	479	Sudden cardiac death
Non-suicidal patients: ratio/ median (q1;q3)		11 m/10 f	54 (46; 62)	48 (20; 52)	18 (9; 25)	280 (111; 740)	50 (30; 52)	135 (85; 321)	
46	Control	m	47	24	–	–	–	311	Acute respiratory failure
47	Control	f	48	48	–	–	–	464	Acute cor pulmonale (pulmonary embolism)
48	Control	m	47	24	–	–	–	616	Coronary failure
49	Control	f	64	24	–	–	–	254	Toxic shock syndrome (sepsis)
50	Control	f	50	72	–	–	–	107	Ruptured abdominal aortic aneurysm
51	Control	m	40	96	–	–	–	205	Acute myocardial infraction
52	Control	m	64	35	–	–	–	63	Sudden cardiac death
53	Control	f	48	26	–	–	–	370	Acute cor pulmonale (pulmonary embolism)
54	Control	f	65	24	–	–	–	348	Sudden cardiac death
55	Control	f	30	48	–	–	–	307	Acute cor pulmonale (pulmonary embolism)
56	Control	f	64	26	–	–	–	178	Sudden cardiac death
57	Control	m	63	48	–	–	–	247	Sudden cardiac death
58	Control	f	61	8	–	–	–	276	Sudden cardiac death
59	Control	f	38	24	–	–	–	176	Sudden cardiac death
60	Control	m	39	4	–	–	–	139	Toxic shock syndrome (peritonitis)
61	Control	f	61	24	–	–	–	61	Sudden cardiac death
62	Control	f	67	24	–	–	–	1191	Sudden cardiac death
63	Control	m	54	24	–	–	–	141	Sudden cardiac death
64	Control	f	63	24	–	–	–	266	Sudden cardiac death
65	Control	f	39	48	–	–	–	71	Exposure to excessive natural heat
66	Control	f	66	24	–	–	–	2484	Sudden cardiac death
67	Control	f	39	48	–	–	–	390	Acute myocardial infarction
Controls: ratio/median (*q*1; *q*3)		7 m/15 f	52 (40; 64)	24 (24; 48)	–	–	–	260 (141; 370)	

Statistic									
Suicidal versus non-suicidal patients versus controls									
Test		*χ²*	*H*	*H*	*–*	*–*	*–*	*H*	
Characteristic value		*χ²* = 1.354	*H* = 3.033	*H* = 0.328	*–*	*–*	*–*	*H* = 2.884	
*P* value		0.508	0.219	0.849	–	–	–	0.236	

Suicidal versus non-suicidal patients									
Test		*χ²*	*U*	*U*	*U*	*U*	*U*	*U*	
Characteristic value		*χ²* = 0.143	*Z* = − 1.582	*Z* = − 0.463	*Z* = − 1.187	*Z* = − 0.905	*Z* = 1.830	*Z* = 0.785	
*P* value		0.905	0.114	0.644	0.239	0.350	0.052	0.436	

Correlation analysis between presented numerical confounding variables and microglia density in the dorsal raphe nucleus									
Group		Age	PMI	Illness duration	CPZ	AD			
Suicidal patients, *r*/*P*		0.28/0.19	−0.04/0.86	0.28/0.25	−0.37/0.47	0.49/0.33			
Non-suicidal patients, *r*/*P*		0.17/0.46	−0.26/0.26	0.23/0.34	0.03/0.94	0.90/0.04			
Controls, *r*/*P*		0.14/0.53	−0.26/0.26						

*Sz* schizophrenia, *MDD* major depressive disorder, *BD* bipolar disorder, *f* female, *m* male, *PMI* postmortem interval, *CPZ* chlorpromazine equivalents of mean daily doses of antipsychotic medication, *AD* amitryptiline equivalents of mean daily doses of antidepressant medication in the last 90 days of life, *q1* and *q3* quartile 1 and 3, *r* correlation coefficient, *P P* value of the Spearman’s correlation

During the last 90 days prior to death, a minority of patients was treated with psychotropic medication. A subset of patients with affective disorder received antidepressant (11 out of 27) and antipsychotic medication (10 out of 27; 7 of them overlapped with those who received antidepressants). A subset of schizophrenia patients (7 out of 18) received antipsychotic medication. Affective disorders and schizophrenia patients who received antipsychotic medication were treated with typical antipsychotic drugs. The mean daily doses of psychotropic medication in the last 90 days of life were established from the clinical records, taking into consideration the equivalents of psychotropic medication present in the references [40–43].

Qualitative neuropathological changes suggestive of vascular, traumatic, inflammatory, neoplastic, and neurodegenerative processes were excluded by an experienced neuropathologist (C. M.). Sections from the prefrontal cortex, the hippocampal complex, the subcortical nuclei, and the brainstem were evaluated in each of investigated cases. No case revealed ischemic foci accompanied by increased microglial reaction. Alterations suggestive of neurodegenerative disorders were excluded by immunostaining for beta-amyloid, hyperphosphorylated tau-protein, and ubiquitin, as well as by Gallyas silver stain. The diagnosis of suicide was established by a forensic pathologist.

The tissue preparation was performed as previously described [4, 24, 39]. Briefly, brains were fixed in toto in 8% phosphate-buffered formaldehyde for at least 2 months (pH = 7.0; temperature 15–20 °C). The brainstem was isolated by a cut made perpendicularly to its longitudinal axis at the point of emergence of the oculomotor nerve. A second transverse cut was made at the caudal level of the medulla. After being embedded in paraffin, serial 20-µm-thick transverse sections were cut along the entire rostrocaudal axis of the brainstem and mounted. Every 50th section was Nissl (cresyl violet) and myelin (Heidenhain-Wölcke) stained. The rostral section of the DRN stained for microglia was adjacent to the one randomly selected from the first three Nissl-stained sections of the rostral DRN at the level of the trochlear nucleus. Accordingly, the caudal section of the DRN stained for microglia was selected at the level of the rostral locus coeruleus. Thus, the selection of sections for microglia staining was in accordance with the principle of systematic sampling. Consequently, one section at the level of the trochlear nucleus containing the ventral, ventrolateral, dorsal, and interfascicular subnuclei, and one section at the level of the rostral locus coeruleus containing the caudal subnucleus of the DRN were used for the evaluation of AgNOR parameters in each of the investigated cases.

### Microglia immunostaining

**Fig. 1:**
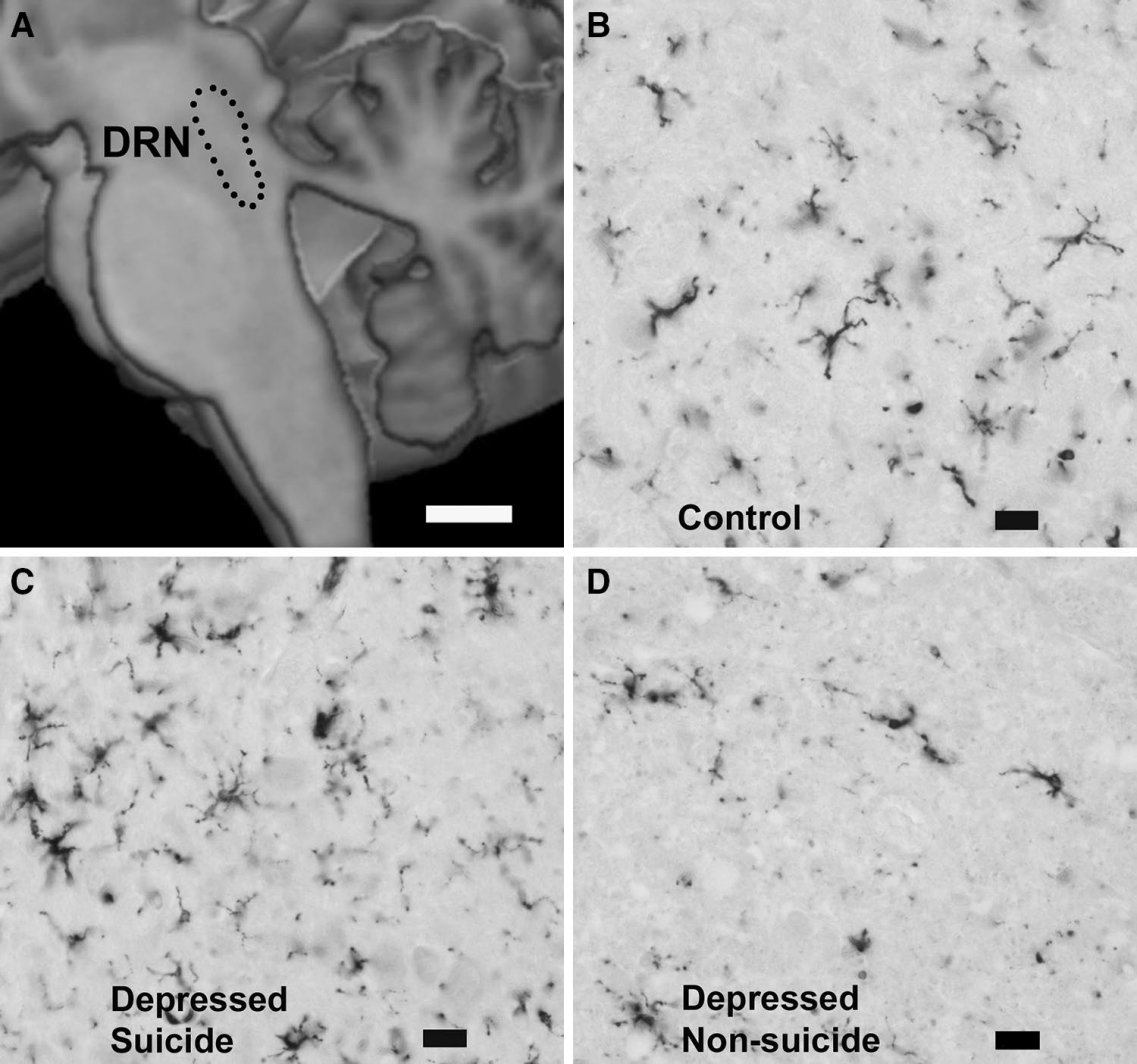
Microglial reaction in the dorsal raphe nucleus (DRN) is decreased in non-suicidal compared to suicidal depressed patients and controls as revealed by the immunostaining for HLA-DR antigen (exemplified by the immunostaining in the ventral subnucleus of the DRN); *scale bars* **a** 10 mm, **b**–**d** 20 µm

Formalin-fixed tissue sections were deparaffinized and treated with 1.5% H_2_O_2_ for 10 min to block endogenous peroxidase activity, followed by blocking of unspecific binding sites with 10% normal goat serum for 60 min. Next, sections were incubated with anti-HLA-DR antibody for 24 h at 4 °C (DAKO Denmark, Clone TAL.1B5, 1:30). Primary antibodies were detected by the Avidin-Biotin-Complex (ABC) method, using biotinylated goat antimouse IgG (Amersham England, RPN 1177, 1:100; 2 h at room temperature) in combination with streptavidin-biotin-peroxidase complex (Amersham England, RPN 1051, 1:100, 1 h at room temperature). The chromogen 3,3΄-diaminobenzidine (DAB) and 0.5% ammonium nickel sulphate hexahydrate were used to visualise the reaction product after a 10 min. incubation at room temperature. The specificity of the HLA-DR antibody has been demonstrated previously [44]. This antibody reacts with the invariant C-terminal tail of HLA-DR [45]. Thus, immunoreactivity is independent of a patient’s HLA-haplotype. Ramified microglia was defined as having thin, radially projecting processes. Ameboid microglia was defined as having densely stained, enlarged cell bodies, and few short processes, if any. Both monocytes and ameboid microglia may have a rounded to oval cell shape. The main criterion in distinguishing between these cells was their location in relation to vessels. Cells visibly located inside vessels were classified as monocytes; cells that were clearly outside of vessels were evaluated as ameboid microglia [46]. Only microglia, i.e., cells located in the parenchyma were evaluated. Representative patterns of microglia immunostaining are shown at Fig. 1b–d.

### Quantification

The regions of interest within anatomical borders of each DRN subnucleus (i.e., the dorsal, ventrolateral, ventral, interfascicular, and caudal, identified according to the description by Baker [47]) were delineated by 200× magnification and delineated areas were measured automatically by computer image analysis system (cellSens® Standard, Olympus, Japan). In each of these five areas, the total number of clearly immunostained microglial cells (i.e., cells located in the parenchyma, which revealed higher staining intensity than background staining) was counted manually (per computer mouse clicks) diagnoses blind by the same magnification (R. B.). Cell densities in each of the DRN subnuclei were calculated by dividing the cell numbers by the measured areas (cells/mm^2^).

The sampled measures were averaged to derive single values for rostral and caudal subdivisions of the DRN and the entire DRN as a single anatomical structure in the investigated individual.

### Data analysis

Statistical analyses were performed with the data analysis software system STATISTICA version 10 (StatSoft®, Inc. 2011, http://www.statsoft.com). As normal distribution (i.e., significant results of the Kolmogorov–Smirnoff test) was not given for the data, non-parametric statistical procedures were used. First, a Kruskal–Wallis analysis of the variance of ranks (*H* test) was performed using the diagnostic group as an independent variable and microglia density as dependent variable. Second, unadjusted two-way post-hoc comparisons with the Mann–Whitney *U* test were carried out to detect between-group differences.

The *χ* ^2^ test, the *U* test, and the *H* test were used to detect the possible differences between the study groups with respect to sex, age (at death and at disease onset), diagnosis (of affective disorders versus schizophrenia), season of the year (month of death in spring/summer versus autumn/winter), postmortem delay, illness duration, medication dosage, brain weight, and fixation time (all statistical tests were two-tailed).

Spearman correlation coefficients were calculated to determine the impact of numerical variables which might confound the dependent variables, i.e., they were also calculated to determine the influence of antidepressants and antipsychotics dosage on the microglia density in depressed patients and antipsychotics dosage on these parameters in schizophrenia patients. Moreover, they were calculated to determine the association between microglia density and AgNOR parameters (investigated previously, [4, 39]) in analysed groups.

In general, *P* values of <0.05 were accepted as statistically significant. When both the *H* test and triple post-hoc comparisons with the *U* test were considered in combination, the *P* values were corrected for multiple comparisons in line with the Bonferroni–Holm–Shaffer procedure.

## Results

### Qualitative analysis of the microglia morphology in the DRN

After immunostaining for HLA-DR antigen, the DRN microglia presented different morphological forms described previously in brains obtained from the Magdeburg Brain Bank [32, 44] (Fig. 1b–d). However, the observed forms were not specifically related to any of the analysed groups or subgroups of patients.

### Quantitative analysis of the microglia density in the DRN

No significant effects specific for both entire diagnostic groups (affective disorders, schizophrenia) and their subgroups (MDD, BD, or paranoid and residual schizophrenia, respectively) were found (non-significant *H* tests and *U* tests *P* values). Similarly, the statistical analysis revealed no significant differences in microglia density between all suicidal and non-suicidal patients from both diagnostic groups and controls by means of the analysis of rostral and caudal subregions of the DRN and the cumulative analysis of all DRN subnuclei. Only non-suicidal depressed patients revealed significantly decreased microglial reaction versus both suicidal depressed patients and controls in the cumulative analysis of the DRN (Table 2).

**Table 2:** Presentation of between-group differences regarding the evaluation of microglia density in the cumulative analysis of dorsal raphe nucleus subnuclei

Group	Microglia density (cells/mm^2^) Median (*q*1, *q*3; *n*)	*H* test *P*	*U* test *P*		
*S*_All_	198 (107, 304; 24)	*S*_All_/NS_All_/*C*	*S*_All_/NS_All_	*S*_All_/*C*	NS_All_/*C*
*S*_D_	196 (126, 280; 16)	n.s	n.s	n.s	n.s
*S*_S_	237 (71, 405; 8)				
		*S*_D_/NS_D_/*C*	*S*_D_/NS_D_	*S*_D_/*C*	NS_D_/*C*
NS_All_	135 (85, 231; 21)	0.016	0.026	n.s	0.025
NS_D_	97 (78, 121; 11)				
NS_S_	286 (135, 480; 10)	*S*_Sz_/NS_Sz_/*C*	*S*_Sz_/NS_Sz_	*S*_Sz_/*C*	NS_Sz_/*C*
		n.s	n.s	n.s	n.s
*C*	260 (141, 370; 22)				

*S* suicidal, *NS* non-suicidal patients (*All* from both diagnostic groups, *D* from affective disorders group, and *Sz* from schizophrenia group), *C* controls, *q1* and *q3* quartile 1 and 3, *n* number of cases, *H test P H* test *P* values, *U test P U* test *P* values corrected for multiple comparisons by Bonferroni–Holm–Shaffer procedure (the level of α remains to be 0.05 after adjustment of *P* values), *n.s*. non-significant

The microglia density in the entire affective disorders group revealed significant correlation with the AgNOR number (*r* = 0.58, *P* = 0.002), which was an effect specific for non-suicidal patients (*r* = 0.62, *P* = 0.04). In the schizophrenia group, this density was correlated with the AgNOR ratio (*r* = 0.51, *P* = 0.04), which was an effect specific for residual patients (*r* = 0.73, *P* = 0.04) (see Table 3). Thus, a positive correlation existed between microglia density and the AgNOR parameter in residual subgroup of schizophrenia patients, which revealed a significant increase in this subgroup in our previous study [39]. No associations between microglia density and AgNOR parameters were found in controls.

**Table 3:** AgNOR parameters of dorsal raphe nucleus neurons, which correlated significantly with microglia density in this nucleus in depressed or schizophrenia patients

AgNOR parameter	*D*	*D*_NS_	*D*_S_	Sz	Sz_res_	Sz_par_
AgNOR number, *r*/*P*	0.58/0.002	0.62/0.04	0.51/0.053	−0.010/0.70	−0.20/0.60	−0.04/0.94
AgNOR ratio, *r*/*P*	0.02/0.91	−0.34/0.31	−0.12/0.67	0.51/0.04	0.73/0.04	−0.18/0.70

*r* correlation coefficient, *P P* value of the Spearman’s correlation, *D* depressed patients, *D*_*NS*_ non-suicidal, *D*_*S*_ suicidal, *Sz* schizophrenia patients, *Sz*_*res*_ residual, *Sz*_*par*_ paranoid

### Confounders

The median daily doses of both antipsychotics and antidepressants given in the last 90 days of life did not differ significantly between suicidal and non-suicidal patients. No significant correlations were found between antipsychotic medication and microglia density in the cumulative analysis of DRN subnuclei in both cohorts. However, a strong positive correlation was found between antidepressants and microglia density in the non-suicide group (Table 1). Therefore, it was an effect which counteracted the observed differences in microglia density (Table 2) taking into consideration that all non-suicidal patients treated with antidepressants (*n* = 5) were depressed patients (Table 1). Moreover, only non-suicidal depressed patients who were untreated with antidepressants (*n* = 6) revealed significantly decreased microglial reaction versus controls (*H* test *P* value 0.042, corrected *U* test *P* value 0.039; for a comparison with the effect obtained for the entire non-suicide depressed subgroup see Table 2). Therefore, the presented decrease in the investigated parameter specific for non-suicidal depressed patients was most probably not confounded by antidepressants.

Neither significant difference was found in numbers of affective disorders and schizophrenia patients between suicide and non-suicide groups (*χ* ^2^ test *P* value 0.329) nor significant intra-group differences in the investigated parameter were found between affective disorders and schizophrenia patients in both suicide and non-suicide groups (non-significant *U* test *P* values).

Similarly, neither significant differences were found in numbers of males and females between compared groups (non-significant *χ* ^2^ test *P* values, Table 1) nor significant differences in microglia density were found between males and females in suicide and non-suicide groups (non-significant *U* test *P* values).

Other potentially confounding variables were neither significantly different between suicide and non-suicide groups nor associated with microglia density (see Table 1 for the analysis of most important confounders).

Almost all non-suicidal patients and controls were sudden death cases (see Table 1). Only one case in the non-suicidal group (Table 1, Case ID 27) and two cases in the control group (Table 1, Cases ID 49 and 60) deceased due to the toxic shock syndrome, which could be possibly related to microglial activation due to prolonged agony, hypoxia, and multiple organ dysfunction. However, none of these three cases revealed extreme values of microglia density in the DRN and the exclusion of them was irrelevant for the statistics. Therefore, it is unlikely that the cause of death (i.e., sudden death versus death with prolonged agony) confounded our results.

## Discussion

Our study revealed a significantly decreased microglial reaction in the DRN in non-suicidal compared with suicidal depressed patients from the affective disorders group and controls. The observed effect was neither confounded by other variables, among them postmortem interval, nor related to psychotropic medication, which partially counteracted this effect. The significance was shown in the cumulative analysis of all DRN subnuclei, i.e., similarly as in our previous AgNOR studies [4, 5, 24, 39]. This phenomenon could be related to the functional characteristics of DRN subregions, as their connections overlap in target structures [7–9] in spite of the accentuated distinctiveness [48, 49]. The overall microglia density in the DRN was higher compared to forebrain regions investigated previously [32], which corresponds with regional differences found in former studies of HLA-DR antigen expression and may be related to different neuroregulatory environments [50]. The observed morphological forms of microglia were not specifically related to any of the analysed groups or subgroups of patients, which corresponds with the previous studies of mental disorders where no significant diagnosis-specific differences in microglia appearance were revealed [32, 33, 51, 52].

In our previous AgNOR study of similar cohorts, we have found that ribosomal DNA (rDNA) transcriptional activity in DRN neurons is decreased in depression compared to schizophrenia [39]. Moreover, the relation between suicide and disturbed rDNA transcription was also differentially accentuated in both diagnostic groups despite existing diagnosis-overreaching similarities [4]. Interestingly, in the current study, we have revealed an association between microglia density and AgNOR number in non-suicidal depressed, but not in non-suicidal schizophrenia patients. Therefore, both our previous AgNOR and present microglia studies of the DRN suggest diagnosis-specific differences, which could be related to differentially disturbed distal afferent inputs (predominantly from the PFC) and local neuronal circuits (reviewed in: [39]).

The interpretation of our current results is not unequivocal. Our previous studies suggested the decreased rDNA transcriptional activity in DRN neurons in depressed suicidal compared to non-suicidal individuals [4, 24]. These results seem to be complementary to our current findings, as the increased microglia activity observed in the DRN of depressed suicides versus non-suicides may result in the decreased rDNA transcription in the former subgroup. As revealed by experimental studies, activated microglia may induce oxidative stress in target neurons (for a review, see: [53]), which, in turn, deteriorates their rDNA transcriptional activity [54]. However, microglia may exert either devastating or restoring effect on neuronal function, which is related to the prevalence of damaging or supportive subpopulations in activated microglia, respectively [13, 55, 56].

Therefore, another interpretation of the increased microglia activity in depressed suicides compared to non-suicides should also be considered. The diminished rDNA transcriptional activity in DRN neurons [4, 5, 24] plays most probably a key role in their deteriorated plasticity in suicide victims [57]. Thus, the observed microglia increase in depressed suicides compared to non-suicides may reflect an attempt for the restoration of decreased neuronal plasticity [56, 58]. Moreover, the increased microglial reaction in depressed suicides compared to non-suicides may constitute rather a consequence than a cause of decreased activity of DRN neurons. Increased HLA-DR antigen presentation was found in microglia as a consequence of the attenuated activity of neighboring neurons in cellular cultures [59], which corresponds with numerous experimental data on the cross-talk between neurons and microglia (for reviews see: [60, 61]). Further research on cytokines produced by microglia in the DRN could possibly help to evaluate the exact pathophysiological relationships between microglia and neurons in suicidal behaviour. However, the increased levels of pro-inflammatory cytokines found in prefrontal regions and cerebrospinal fluid of suicide victims suggest rather the devastating neurodegenerative role of microglia activation in suicide (for reviews, see: [12, 29]).

Therefore, the decreased microglial reaction in the non-suicidal depressed subgroup might rather be interpreted as a suicide-preventive effect. Moreover, microglia density correlated positively with rDNA transcriptional activity in DRN neurons in this subgroup. Similar correlation existed in the residual subgroup of our schizophrenia cohort. Recently, the neuroimaging study of predominantly euthymic BD patients has also suggested positive correlation between microglial reaction and neuronal function [62]. As revealed in cellular cultures, microglia may produce neurotrophic factors ([63]; for a review, see: [55]), which, in turn, stimulate rDNA transcription in neurons ([64]; for a review, see: [65]). The decreased microglial activity in brain structures was revealed in the neuroimaging study of mild to moderately depressed patients [66]. The diminished microglial reaction was also found in the animal model of depression in the hippocampus paralleled by depressive-like behaviour, which was reversed after microglia stimulation by the systemic administration of bacterial endotoxin [67]. This regimen exerted an antidepressant effect in the melancholic subgroup of depressed patients [68], which was related to the microglia activation according to current neuroimaging data [69]. Taking together, these findings suggest the restorative function of microglia in non-suicidal depressed individuals in opposite to their neurodegenerative or disturbed restorative roles in depressed suicide victims from our cohort.

The strong positive correlation between antidepressant medication and HLA-DR positive microglia density found in non-suicidal patients supports the presented hypothesis on the protective role of microglial reaction in this subgroup. Both animal models [67, 70, 71] and human data [71, 72] suggest the involvement of adequate microglial activity in successful antidepressant treatment. Therefore, our results support the concept of personalised treatment of abnormal microglial reaction in depression, i.e., the treatment aimed at either augmentation or attenuation of this reaction according to individually assessed immunological status of the patient [13, 67, 72].

## Limitations

This study has certain limitations that have to be considered: (1) a relatively small number of cases have been analysed, especially for the evaluation of schizophrenia patients; therefore, results have to be confirmed in a larger sample. (2) A long-term influence of antidepressants and antipsychotics on the outcome of our study cannot be excluded, because no reliable data on this medication for a period beyond the 3 months prior to death were available. (3) Moreover, the relatively small number of treated patients prevents from a conclusive statement whether antipsychotics and antidepressants did, in fact, influence microglia density in the DRN.

## Conclusion

In summary, we have revealed an abnormal microglial reaction in the DRN restricted to the affective disorders cohort. The results suggest region- and diagnosis-specific differences in this reaction compared to our previous study of forebrain regions in suicide [32]. The diminished activation of microglia in the DRN is a phenomenon specific for non-suicidal depressed patients, where also a positive association seems to exist between antidepressant medication, microglia activation, and rDNA transcription. The results suggest a possible suicide-preventive effect of microglial reaction restricted to this subgroup, whereas an opposite effect may exist in depressed suicidal patients. However, further research on cohorts containing more numerous suicidal and non-suicidal cases with different psychiatric diagnoses is warranted to appropriately evaluate this issue.
